# A study on Mahjong intervention to improve cognitive impairment in patients with schizophrenia: a pilot, single-blind, randomized, controlled trial

**DOI:** 10.1186/s12888-025-07321-1

**Published:** 2025-11-07

**Authors:** Renqin Hu, Zongli Xie, Junyao Li, Huirong Luo, Yanwei Guo, Jinglan Tan, Qinghua Luo

**Affiliations:** 1 Department of Psychiatry, the First Affiliated Hospital of Chongqing Medical University, Chongqing, 400016 China; 2 Department of Psychiatry, Yubei District Hospital of Traditional Chinese Medicine, Chongqing, China

**Keywords:** Schizophrenia, Mahjong, Cognitive function, Quality of life

## Abstract

**Objective:**

Mahjong, a traditional Chinese tile-based game, has been widely reported to be closely associated with better cognitive function. However, its effects on the cognitive function of patients with schizophrenia have not yet been studied.

**Method:**

In a pilot study, 49 patients diagnosed with schizophrenia were randomly assigned to the intervention group (Mahjong combined with standard treatment) and the control group (standard treatment). The intervention group engaged in cognitive training through Mahjong for 2 h per day, 4 days per week for 12 weeks. Primary cognitive outcomes were assessed using Cambridge Neuropsychological Test Automated Battery (CANTAB), while secondary outcomes include quality of life, clinical symptoms, anhedonia, treat side effects, and personal and social functioning. Assessments were conducted at baseline (T0), the 4th week (T1), the 8th week (T2), and the 12th week (T3).

**Results:**

The intervention group exhibited progressive improvements in both reaction time and movement time throughout the study. No significant differences were found between the intervention and control groups regarding visual memory, novel learning, strategy utilization, spatial memory performance or complex visual task accuracy. The intervention group demonstrated gradual improvements in quality of life, whereas no significant changes were noted in other secondary outcomes.

**Conclusion:**

While this exploratory study suggests that Mahjong intervention may benefit certain cognitive functions and quality of life in patients with schizophrenia, these findings should be interpreted with caution. Further research with larger, more diverse samples and longer intervention is necessary to confirm and extend these findings.

**Trial registration:**

The trial is registered with https://www.chictr.org.cn/ under registration number ChiCTR2400080268 on January 25th, 2024.

**Supplementary Information:**

The online version contains supplementary material available at 10.1186/s12888-025-07321-1.

## Introduction

Mental disorders remain one of the top ten global burdens, and schizophrenia has one of the highest measured years lived with disability among all diseases, injuries, and risk factors [1]. Cognitive deficits have long been considered a core symptom of schizophrenia and are significant factors leading to disability and treatment refractoriness in schizophrenia patients [2, 3]. These deficits are pervasive, affecting processing speed, verbal memory, working memory, attention, and executive functions [2, 4, 5]. Various cognitive deficits in schizophrenia are closely related to functional outcomes and quality of life [6], posing major obstacles to maintaining work and social life [5, 7]. Therefore, improving cognitive deficits in schizophrenia has garnered increasing attention [8].

Despite significant achievements in treating acute and maintenance phases of schizophrenia [9–11], cognitive deficits have not been adequately addressed [12], with only a few antipsychotic drugs showing potential cognitive benefits [13]. Moreover, the molecular targets for treating cognitive deficits mentioned in the MATRICS have not been sufficiently proven to have cognitive benefits [14–17]. The adjunctive use of cognitive enhancers for schizophrenia patients has shown limited positive effects and lacks evidence to support long-term use [18, 19]. Even promising treatments like transcranial direct current stimulation still require more refinement in therapeutic parameters for restoring cognitive function in schizophrenia patients [20, 21].

Cognitive remediation (CR), a widely proven treatment to improve cognitive function [22, 23], but efforts continue to promote CR as a routine treatment for schizophrenia patients [24]. Trained therapists, cognitive exercises, development of cognitive strategies, and facilitating the transfer of cognitive gains to daily functioning are vital factor in the success of CR in producing a cognitive effect [22, 25]. Even relatively more convenient computerized CR still requires supplementary human guidance to achieve greater cognitive benefits [26]. However, not all regions have enough trained psychiatrists [27]. This issue is more pronounced in Asian regions, especially in underdeveloped areas [28–32]. Additionally, the COVID-19 pandemic has further increased the healthcare burden [33]. These problems have significantly hindered the implementation of CR. Therefore, finding an adjunctive treatment that is beneficial for cognitive function and has low healthcare burden is of great importance.

Mahjong is a popular social entertainment in China with a gambling nature. The Chinese Longitudinal Healthy Longevity Survey has demonstrated that Mahjong is closely associated with cognitive improvement among the elderly population [34], with higher frequencies of play correlating with better cognitive outcomes [35, 36]. Randomized controlled trials (RCT) involving individuals with cognitive impairments or dementia have indicated that Mahjong can enhance attention, verbal memory, and executive function [37–39]. However, due to the lack of research focusing on patients with schizophrenia [40], it remains unclear what effects Mahjong may have in this population. Mahjong serves as a cognitive exercise that incorporates both bottom-up and top-down approaches. On one hand, it facilitates training of low-level sensory, perceptual, and cognitive processes; on the other hand, it engages high-level memory processes, reasoning, problem-solving, executive function and metacognition [41]. Moreover, the inherently entertaining nature of Mahjong makes it intrinsically engaging. Therefore, Mahjong holds promise as a potentially effective method for improving cognitive function [42].

Importantly, Mahjong does not require professional medical personnel for guidance and participation; it can be organized and maintained by the players themselves, which may facilitate its integration into institutional daily routines with minimal additional cost [38]. Otherwise, Mahjong, as a form of social participation, plays a role in increasing interpersonal communication, social support, and promoting mental health [43–47], which helps alleviate stress-related neuronal changes and improve cognitive function [48, 49].

## Objective

This study reports a preliminary RCT testing the hypothesis that Mahjong intervention in the daily lives of patients with schizophrenia can improve their cognitive function, quality of life, and clinical outcomes.

## Method

### Study design

**Fig. 1:**
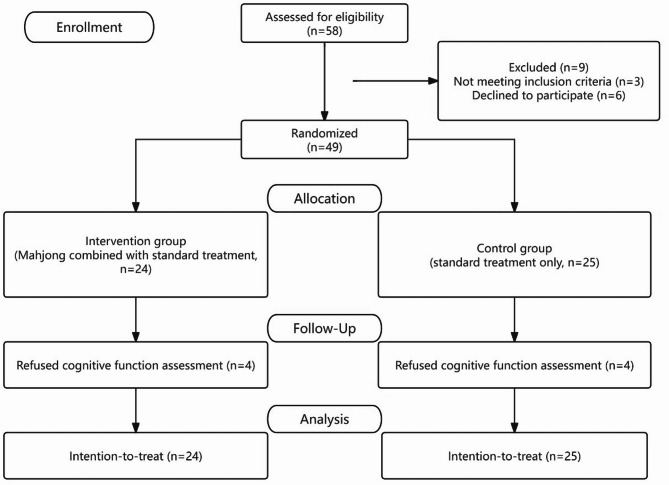
CONSORT diagram

This pilot, single-blind, RCT was approved by the research ethics committee of the First Affiliated Hospital of Chongqing Medical University (Approval Number: 2024-010-02) and registered with the China Clinical Trials Center (Registration Number: ChiCTR2400080268, Date of Registration: 2024-01-25). Figure 1 represents the research procedure schematically. Notably, the initial protocol described a non-randomized, preference-based allocation (1:2 ratio) to account for anticipated dropout in the intervention group. However, during the preparatory phase, participants showed unexpectedly high interest and adherence. To improve internal validity and reduce potential bias, we revised the design to a 1:1 randomized controlled trial before group allocation and data collection. The updated protocol was approved by the ethics committee.

### Participants and enrollment

Participants were recruited from the Department of Psychiatry, Yubei District Hospital of Traditional Chinese Medicine, Chongqing, and lived long-term in the inpatient department, with relatively uniform schedules and had sufficient time to play Mahjong. As the trial was conducted in the male patient ward, female patients were not included in this study. Participants were recruited starting in February and ending in May 2024. During the study period, patients showed no significant changes in symptoms, allowing them to continue with maintenance medication regimens.

Eligible participants were over 18 years old, with sufficient ability to understand how to win at Mahjong and complete cognitive tests. They were diagnosed with schizophrenia by two psychiatrists according to DSM-5 [50], had clinically stable conditions with no recent changes in maintenance medication, and a total PANSS score below 60. Patients were excluded if they had severe physical illnesses, organic brain diseases, a history of head trauma, substance abuse, or exhibited manic, impulsive, or uncooperative behavior.

Inpatients were introduced to this activity as a welfare program organized by healthcare staff, providing with the space and Mahjong equipment. A total of 58 patients expressed interest in participating. They received training on Mahjong rules to ensure they understood the combinations necessary to win and the types of combinations representing higher-value wins, regardless of their prior Mahjong experience. During this process, 3 patients were excluded for being unable to grasp the rules, and 6 withdrew consent, as they declined to adhere to the intervention schedule. After obtaining informed consent from patients and their families, a total of 49 patients were included in the study.

### Randomization

After completing Mahjong training, participants were randomly assigned to 2 groups. The intervention group received Mahjong combined with standard treatment, while the control group received only standard treatment. A statistician, independent of other trial procedures, generated the randomization sequence using Microsoft Office Excel 2017. The randomization sequence was concealed from investigators, and allocation concealment was maintained through consecutively numbered, opaque, sealed envelopes. To ensure objectivity and minimize bias, a research nurse, uninvolved in other study activities, assigned participants to their respective groups.

Due to the nature of the intervention, instructors and participants could not be blinded to the group assignments. To minimize potential bias, assessors and data analysts were kept unaware of the intervention groups throughout the study.

### Sample size

As this is the first study investigating Mahjong intervention for schizophrenia, no directly comparable trials were available for estimation. Thus, we referred to previous randomized controlled trials targeting cognitive improvement in patients with schizophrenia [51, 52], and Mahjong interventions in populations with cognitive impairments [37–39], to guide our recruitment strategy. Based on these precedents, a total sample size of 49 participants was deemed acceptable for this pilot trial.

Due to changes in study design after trial initiation, we were unable to conduct a valid priori power analysis. Therefore, only post-hoc power analyses were conducted using PASS 15.0 software. Based on the actual sample sizes (*n* = 24 and 25), four repeated measurements, an alpha level of 0.05, and the observed means for each outcome, statistical power was estimated for the 11 primary and secondary outcomes. Nine outcomes showed adequate power (0.50–1.00), while two outcomes (RTI five-choice accuracy score and SQLS total score) showed relatively low power (0.30). Overall, the study had sufficient power to detect intervention effects for most outcomes, though caution is warranted in interpreting those with limited power.

### Assessment

The first assessment (T0) was conducted prior to randomization, with subsequent assessments conducted at the fourth week (T1), eighth week (T2), and twelfth week (T3), corresponding to the end of the intervention period.

#### Cognitive function

The Cambridge Neuropsychological Test Automated Battery (CANTAB) is a computerized, non-verbal neuropsychological assessment tool designed to minimize language and cultural bias. It has shown strong reliability and validity in assessing cognitive function in schizophrenia [53], with well-established sensitivity [54]. Mahjong, as a cognitively demanding activity, involves monitoring others’ actions, retaining visual information, and engaging in rapid pattern recognition and decision-making. These cognitive domains are closely aligned with the core cognitive deficits in schizophrenia, particularly in processing speed, working memory, and visual memory [55]. Therefore, we selected the following three CANTAB subtests to assess these domains:

1. Reaction time (RTI) measure the subject’s speed of response to a visual target where the stimulus is either predictable (simple reaction time) or unpredictable (choice reaction time). The outcome measures include accuracy score, reaction time and movement time.
2. Spatial working memory (SWM) assesses the subject’s ability to retain and manipulate spatial information in working memory. The outcome measures include between errors and strategy employed. A high strategy score represents poor use of strategy, and a low score equates to effective use.
3. Paired associates learning (PAL) assesses visual memory and new learning. The subject is required to remember randomized patterns and accurately identify their original locations. The outcome measures include number of errors.

#### Clinical outcome

Clinical outcomes included Positive and Negative Syndrome Scale (PANSS) [56], Clinical Global Impression-Severity (CGI-S) [57], Self-report Quality of Life Measure for People with Schizophrenia (SQLS) [58], Personal and Social Performance Scale (PSP) [59], Treatment Emergent Symptom Scale (TESS) [60], Snaith-Hamilton Pleasure Scale (SHAPS) [61], and Dimensional Anhedonia Rating Scale (DARS) [62].

### Intervention

Considering that repetitive cognitive exercise can produce greater cognitive benefits [23, 27], the intervention group engaged in cognitive training through Mahjong for 2 h per day, 4 days per week for 12 weeks. During the trial, researchers reminded and arranged for players, and subjects could also organize their own Mahjong sessions.

Mahjong is typically played by four participants seated at a square table, representing the compass points of east, south, west, and north. The game utilizes a set of 144 tiles, with each player initially dealt thirteen tiles from the tile wall, while the dealer, seated in the east position, receives fourteen tiles. The objective of the game is to create a winning hand consisting of four sets of three tiles and a pair of tiles of the same suit, totaling fourteen tiles. The sets can comprise either three identical tiles (known as a “pung”) or three consecutive titles in the same suit (known as a “chow”). Victory is achieved when a player forms a complete hand and declares “Mahjong”. This declaration signals the end of the game, and the winning player reveals their hand to calculate points based on the hand’s complexity and any predetermined scoring rules. Due to the patients’ cognitive impairments, we chose the simplest Mahjong rules, where the suits only consist of three types: bamboos, characters, and dots. Detailed rules can be found in The Mahjong Guide – Learn how to play mahjong [63].

### Statistical analysis

Data analysis was conducted using SPSS 25.0 statistical software. Quantitative data were expressed as mean ± SD if approximately normally distributed, otherwise as median and IQR. Intention-to-treat (ITT) analysis was conducted with all randomly assigned participants included in the analysis. Continuous variables were compared using Student’s t-test or Mann-Whitney U test. Considering the non-independence of data and the non-normal distribution of some outcomes, Generalized Estimating Equation (GEE) model was applied. During the modeling process of the GEE, interaction terms between time and group were introduced. *P*-value of less than 0.05 was considered statistically significant.

To control for potential confounders, we adjusted for covariates including age, education, disease duration, PANSS total score, and antipsychotic dose equivalents. Patients’ medication regimens were converted to olanzapine-equivalent doses for comparison purposes [64]. For cognitive outcomes, their baseline scores were also included as covariates. For clinical outcomes, age, education, and disease duration were adjusted.

To address missing data in our study, Last Observation Carried Forward (LOCF) and Complete Case Analysis were employed. To assess the robustness of our data analysis, we performed GEE analysis on both the LOCF and complete case datasets and compared these results with those from the ITT dataset. All covariates used in the analyses remained unchanged across different datasets.

## Result

### Baseline characteristics of participants

Participants in the intervention and control groups did not differ in demographic, cognitive, and clinical characteristics (Table 1). During the study, 8 patients dropped out (4 in intervention group, 4 in control group), while 41 patients completed the trial. Comparing the baseline characteristics between dropout and completer patients revealed that dropout patients were older (50.88 years vs. 42.10 years, *p*<0.001), had a longer duration of illness (22.38 years vs. 17.54 years, *p* = 0.001).

**Table 1:** Baseline demographic, cognitive and clinical characteristics of participants

Variable	Intervention (*n* = 24)	Control (*n* = 25)		*P* value
Age, years^a^	42.3 (8.2)		44.7 (7.5)	0.301
Education, years^b^	8.5 (3.0)		9.0 (1.0)	0.395
Duration of disease, years^a^	17.4 (6.3)		19.2 (7.0)	0.355
Antipsychotic drug doses, mg/d^b c^	8.0 (8.4)		9.8 (5.8)	0.703
PAL Total errors (adjusted)^b^	48.0 (78.0)		67.0 (44.0)	0.167
PAL Total errors (6 shapes, adjusted)^b^	16.5 (37.0)		28.0 (23.0)	0.207
SWM Between errors^b^	44.5 (40.0)		64.0 (28.0)	0.211
SWM Strategy^b^	40.0 (10)		43.0 (8.0)	0.434
RTI Simple accuracy score^b^	14.1 (1.0)		14.3 (0.8)	0.948
RTI simple reaction time^b^	457.7 (217.9)		485.7 (189.6)	0.631
RTI simple movement time^a^	654.6 (191.9)		697.4 (187.9)	0.435
RTI Five-choice accuracy score^b^	14.0 (2.0)		14.0 (2.0)	0.958
RTI Five-choice reaction time^b^	517.5 (209.1)		539.5 (211.3)	0.841
RTI five-choice movement time^a^	729.4 (170.6)		737.3 (181.0)	0.876
Total SQLS score^a^	53.3 (10.6)		48.2 (13.3)	0.141
Total PANSS score^a^	48.8 (5.3)		50.0 (6.1)	0.478
CGI-S^b^	2.0 (1.0)		2.0 (1.0)	0.610
TESS^a^	7.8 (4.4)		7.2 (3.4)	0.468
PSP^b^	69.0 (5.0)		70.0 (5.0)	0.414
SHAPS^a^	28.0 (4.5)		27.9 (5.1)	0.955
DARS^a^	41.8 (10.8)		40.4 (11.5)	0.656

*CGI-S* Clinical Global Impression-Severity, *DARS* Dimensional Anhedonia Rating Scale, *PAL* Paired Associates Learning, *PANSS* Positive and Negative Syndrome Scale, *PSP* Personal and Social Performance Scale, *RTI* Reaction Time, *SHAPS* Snaith-Hamilton Pleasure Scale, *SQLS* Self-report Quality of Life Measure for People with Schizophrenia, *SWM* Spatial Working Memory, *TESS* Treatment Emergent Symptom Scale

^a^Mean (SD)

^b^Median (IQR)

^c^All antipsychotic doses were appropriately converted to olanzapine doses [64]

### Cognitive function outcomes

Both RTI simple and five-choice reaction times showed significant group-by-time interaction effects (Wald χ² = 18.162, *p* < 0.001; 19.944, *p* < 0.001) and significant time effects (Wald χ² = 9.161, *p* = 0.027; 11.184, *p* = 0.011), with no significant group effect for either test. The intervention group exhibited a continuous reduction in movement time across sessions, implying an improvement in motor speed within the intervention group.

For movement time, both RTI simple and five-choice tests showed significant group-by-time interaction effects (Wald χ² = 15.532, *p* = 0.001; 24.436, *p* < 0.001) and significant time effects (Wald χ² = 36.071, *p* < 0.001; 34.482, *p* < 0.001), with no significant group effect. The intervention group showed a reduction in movement time, indicating improved motor speed.

The RTI simple accuracy score showed a significant group-by-time interaction (Wald χ² = 8.176, *p* = 0.043), and the PAL total errors (adjusted) showed a significant main effect of time (Wald χ² = 8.234, *p* = 0.041). However, the RTI five-choice accuracy score and the PAL total errors (6 shapes, adjusted) showed no significant effects. These findings collectively imply that the 12-week intervention may have a limited impact on improving accuracy, visual memory, and novel learning in more complex tasks.

Regarding SWM strategy and between errors, no significant effects were observed, indicating no significant differences in the utilization of strategies or spatial memory performance between groups during the study (Table 2).

**Table 2:** Primary and secondary outcomes

Outcome	Intervention (*n* = 24)	Control (*n* = 25)	Group-by-Time Interaction Effect		Time effect		Group effect	
			Wald χ2	*P*	Wald χ2	*P*	Wald χ2	*P*
RTI Simple accuracy score			8.176	0.043	4.810	0.186	3.281	0.070
T0	14.17 (0.98)	14.20 (0.85)						
T1	14.33 (0.80)	13.46 (1.32)						
T2	14.19 (0.90)	13.62 (1.52)						
T3	14.36 (0.90)	14.03 (1.64)						
RTI simple reaction time, ms			18.162	<0.001	9.161	0.027	1.646	0.199
T0	492.58 (166.90)	515.75 (166.84)						
T1	484.71 (252.44)	511.67 (144.34)						
T2	463.34 (155.85)	561.97 (232.73)						
T3	425.91 (135.79)	533.17 (185.18)						
RTI simple movement time, ms			15.532	0.001	36.071	<0.001	2.695	0.101
T0	654.61 (187.83)	697.36 (184.05)						
T1	613.22 (145.97)	653.51 (165.66)						
T2	594.79 (153.28)	675.71 (137.17)						
T3	544.76 (123.18)	662.90 (150.50)						
RTI Five-choice accuracy score			1.554	0.670	5.669	0.129	0.725	0.394
T0	13.88 (0.89)	13.84 (0.97)						
T1	13.96 (1.14)	13.50(1.57)						
T2	14.09 (1.45)	13.92(1.28)						
T3	14.22 (1.41)	13.91(1.29)						
RTI five-choice reaction time, ms			19.944	<0.001	11.184	0.011	0.729	0.393
T0	553.33 (183.05)	545.26 (159.34)						
T1	539.98 (275.81)	543.96 (147.32)						
T2	509.36 (162.11)	583.59 (202.72)						
T3	460.82 (136.49)	561.85 (196.20)						
RTI five-choice movement time, ms			24.436	<0.001	34.482	<0.001	0.858	0.354
T0	729.39 (167.05)	737.25 (177.35)						
T1	683.17 (150.43)	686.87 (169.57)						
T2	667.91 (175.50)	717.36 (155.42)						
T3	604.34 (144.18)	710.93 (165.40)						
SWM Between errors			4.828	0.185	3.717	0.294	3.460	0.063
T0	48.50 (23.38)	56.92 (20.17)						
T1	45.13 (23.20)	58.87 (22.26)						
T2	45.34 (22.04)	56.87 (21.08)						
T3	44.93 (19.10)	56.43 (20.25)						
SWM Strategy			5.722	0.126	1.003	0.800	0.030	0.862
T0	38.17 (7.65)	39.52 (8.40)						
T1	39.13 (7.01)	37.81 (8.60)						
T2	38.88 (6.12)	39.03 (9.12)						
T3	38.40 (5.70)	39.64 (7.95)						
PAL Total errors (adjusted)			2.834	0.418	8.234	0.041	0.496	0.481
T0	68.46 (55.76)	75.60 (44.13)						
T1	63.29 (51.31)	75.59 (43.17)						
T2	62.09 (44.40)	71.59 (45.63)						
T3	64.01 (45.17)	73.60 (45.37)						
PAL Total errors (6 shapes, adjusted)			0.532	0.912	4.067	0.254	0.770	0.380
T0	25.25 (17.68)	29.60 (13.65)						
T1	25.00 (17.66)	28.45 (13.85)						
T2	24.47 (15.08)	27.86 (13.68)						
T3	24.28 (14.60)	28.06 (14.27)						
SQLS total score			12.042	0.007	23.847	<0.001	0.008	0.931
T0	53.34(10.34)	48.20(13.00)						
T1	49.30(11.51)	47.41(13.13)						
T2	44.11(10.59)	45.97(14.59)						
T3	43.30(9.73)	47.38(12.95)						

Data are estimated marginal means (SD), derived from GEE models adjusted for covariates

*PAL* Paired Associates Learning, *RTI* Reaction Time, *SQLS* Self-report Quality of Life Measure for People with Schizophrenia, *SWM* Spatial Working Memory

### Clinical outcomes

The SQLS total score showed a significant group-by-time interaction (Wald χ² = 12.042, *p* = 0.007) and effect of time (Wald χ² = 23.847, *p* < 0.001), revealed improved quality of life for the intervention group (Table 2). No significant effects were found for other clinical outcomes, including PANSS, CGI-S, SHAPS, DARS, TESS, and PSP, suggesting limited impact on psychotic symptoms, anhedonia, treat side effects, and personal/social functioning.

### Sensitivity analysis

The sensitive analysis results indicated that the statistical significance of the group-by-time interaction, time effect, and group effect were consistent across the ITT, LOCF, and complete case datasets, demonstrating the robustness of our primary findings.

## Discussion

To the authors’ knowledge, this is the first RCT aimed at exploring the effects of Mahjong intervention on cognitive function in patients with schizophrenia. Over time, the intervention group showed improvements in reaction time and movement time of response to a visual target. Moreover, the quality of life of schizophrenia patients also improved during the Mahjong intervention. However, the 12-week intervention did not result in significant improvements in accuracy, visual memory, or novel learning for complex cognitive assessment. Furthermore, the Mahjong intervention demonstrated no discernible effect on broader clinical outcomes.

Previous research indicates that playing Mahjong can provide cognitive benefits for the elderly [65] and is associated with a slower decline in cognitive function [36, 66]. Even among individuals already diagnosed with dementia, Mahjong can yield cognitive benefits, and these effects persist for up to a month after stopping the activity [38]. Additionally, similar to other cognitive games, playing Mahjong involves various cognitive functions. Participants need to analyze and strategize based on the information they receive, followed by logical operations, thus gaining cognitive training in the process [67, 68]. Our study found that after Mahjong intervention, schizophrenia patients showed significant improvement in reaction time, consistent with previous research indicating that Mahjong can enhance attention [69]. Mahjong requires attentional control to quickly identify and remember visual information, while patients’ eye-hand coordination is repeatedly trained [70], ultimately leading to improvements in specific cognitive functions.

Research has also preliminarily explored the brain changes induced by Mahjong. For instance, during Mahjong, an increase in oxygenated hemoglobin concentration in language-related cortical areas has been observed [71], and identifying Mahjong tiles by touch activates the player’s bilateral sensorimotor cortex [72]. Similarly, in experts who have played shogi (Japanese chess) for a long time, neural response times in the frontal and temporal lobes are significantly shortened, allowing quicker reactions to global and local cognitive information [73]. Additionally, early studies have found that repetitive motor tasks in healthy individuals can lead to motor cortex activation and behavioral improvements [74]. Like other cognitive activity, in processing complex information such as in Mahjong, the brain utilizes neurons distributed across multiple processing levels and regions to complete relevant perceptual and cognitive inputs and action outputs [42]. This type of board game appears to meet the three key points of neuroscience-informed cognitive training methods, namely enhancing perceptual processes, ensuring intensive and adaptive training schedules, and engaging the brain’s reward and attention systems [42]. These factors might explain why Mahjong intervention improves cognitive function in schizophrenia patients.

In addition to cognitive impairments and clinical symptoms, social isolation can significantly impact patients’ quality of life [75]. Mahjong, as a group social recreational activity, is often associated with better quality of life in healthy populations [47, 76]. Participation in recreational activities can also benefit people with psychosis [77], and the frequency of participation is positively correlated with better quality of life [78]. Previous studies have shown that Mahjong can reduce depressive symptoms in patients with mild dementia [79], and most psychosocial interventions can improve the quality of life for patients [80]. In this trial, we found that patients showed improvements in SQLS after participating in Mahjong intervention. This improvement could be because leisure activities impart their mental health benefits by increasing individuals’ perception of spending their time effectively [81]. In addition, as the social motivation theory suggests, humans are driven by the need to belong and be socially connected. When schizophrenia patients engage in Mahjong, their fundamental psychological needs are met, which can enhance overall well-being and happiness.

Given that schizophrenia patients’ cognitive function and brain structure deteriorate over time [82, 83]. Early prevention of progressive cognitive decline in schizophrenia patients may be necessary [84]. However, due to economic and resource constraints, priority and awareness issues, personalization and suitability deficiencies, and the lack of implementation context and evidence, CR struggles to become a routine cognitive treatment method [24]. Therefore, before CR can be widely implemented, incorporating board games like Mahjong, which have cognitive training and social attributes, low medical burden, and can be conducted independently by patients, into the daily activities of schizophrenia seems to have potential benefits, especially for long-term hospitalized patients.

The lack of significant improvement in certain cognitive domains when comparing the two groups could be due to the intensity and specificity of cognitive training required for these complex functions [85], which may not be fully addressed by playing Mahjong. Therefore, cognitive training that does not specifically target working memory, is unlikely to produce significant transfer effects [86]. Additionally, reduced gray matter volume [87], white matter disruptions [88] and excessive synaptic deficits [89] collectively contribute to the limited efficacy of Mahjong interventions.

However, playing Mahjong may have potential side effects for participants. For example, prolonged sitting is associated with several pathophysiological changes, including decreased cardiorespiratory fitness and impaired vascular function [90, 91]. Additionally, the risk of metabolic syndrome due to long-term antipsychotic medication use may exacerbate these adverse effects [92, 93]. Furthermore, the gambling aspect of Mahjong may increase the risk of addiction [94]. Excessive gambling can deplete personal savings [95], leading to financial instability and increased economic stress [96].

It is also important to note that Mahjong is deeply embedded in Chinese cultural traditions, with specific rules, terminology, and symbols that may not be easily understood or culturally acceptable in other populations [97]. To enhance cultural adaptability and global relevance, future studies may consider analogous, low-cost cognitive activities, like bridge—commonly played in Western contexts—or chess, which has broad international appeal. Such adaptations would improve cultural and linguistic accessibility, thereby enhancing the global relevance and clinical utility of cognitive interventions.

### Limitation

As a pilot trial, several limitations should be acknowledged. Firstly, this was a single-center study with a small sample size. The findings may not be representative of the population of patients with different states of schizophrenia. Secondly, due to practical constraints, our study included only male patients. This gender-specific sample may introduce a bias and limit the applicability of our findings to female patients. Previous research has shown that gender differences can influence cognitive function in schizophrenia [98]. Thirdly, our sample predominantly consisted of patients with long disease durations and older age. Chronicity and age can significantly impact cognitive function and plasticity, potentially affecting the responsiveness to cognitive interventions [99]. Fourthly, variations in prior Mahjong experience among participants and non-standardized Mahjong training may have introduced biases into the study. However, the randomized controlled design of this study may have helped mitigate such differences. Additionally, patients who showed an interest in Mahjong were enrolled. This selection criterion could introduce a self-selection bias, as patients who are naturally inclined towards engaging in such cognitive activities might already have better baseline cognitive function or motivation, potentially skewing the results. Lastly, our cognitive assessment did not include measures of executive function or other core cognitive deficits in schizophrenia, which may have limited the detection of Mahjong’s potential effects on higher-order cognition.

## Conclusion

In conclusion, while this pilot study suggests that Mahjong training may benefit processing speed, motor speed and quality of life in patients with schizophrenia, further research with larger, more diverse samples and longer intervention is necessary to confirm and extend these findings.

## Supplementary Information

Supplementary Material 1.

Supplementary Material 2.

Supplementary Material 3.

## Data Availability

The datasets used and analyzed during the current study are available from the corresponding author on reasonable request.

## Abbreviations

CANTAB: Cambridge Neuropsychological Test Automated Battery
CR: Cognitive remediation
GEE: Generalized Estimating Equation
ITT: Intention-to-treat
LOCF: Last Observation Carried Forward
PAL: Paired associates learning
PANSS: Positive and Negative Syndrome Scale
RTI: Reaction time
SQLS: Self-report Quality of Life Measure for People with Schizophrenia
SWM: Spatial working memory
