# Supplementary material for: A study on Mahjong intervention to improve cognitive impairment in patients with schizophrenia: a pilot, single-blind, randomized, controlled trial
Source: BMC Psychiatry. 2025 Nov 7;25:1070. doi: 10.1186/s12888-025-07321-1 (PMC12595832; doi:10.1186/s12888-025-07321-1)
Supplement: Supplementary file 2 — Supplementary Material 2. [file 12888_2025_7321_MOESM2_ESM.docx]

**Statistical analysis**

Data analysis was conducted using SPSS 25.0 statistical software. Quantitative data were expressed as mean ± SD if approximately normally distributed, otherwise as median and IQR. Intention-to-treat (ITT) analysis was conducted with all randomly assigned participants included in the analysis. Continuous variables were compared using Student's t-test or Mann-Whitney U test. Considering the non-independence of data and the non-normal distribution of some outcomes, Generalized Estimating Equation (GEE) model was applied. During the modeling process of the GEE, interaction terms between time and group were introduced. For the cognitive outcome, adjustments were made for age, education, disease duration, dose equivalents for olanzapine, PANSS total score, and their respective baseline results. For clinical outcomes, adjustments were made for age, educational level, disease duration. *P*-value of less than 0.05 was considered statistically significant.

To address missing data in our study, Last Observation Carried Forward (LOCF) and Complete Case Analysis were employed. To assess the robustness of our data analysis, we performed GEE analysis on both the LOCF and complete case datasets and compared these results with those from the ITT dataset. All covariates used in the analyses remained unchanged across different datasets.

| **Table 2.** Clinical Outcomes | | | | | | | | |
| --- | --- | --- | --- | --- | --- | --- | --- | --- |
| Outcome | Intervention (n=24) | Control  (n=22) | Group-by-Time Interaction Effect | | Time effect | | Group effect | |
|  |  |  | Wald χ2 | *P* | Wald χ2 | *P* | Wald χ2 | *P* |
| PANSS total score | | | 7.518 | 0.057 | 1.754 | 0.625 | 1.239 | 0.266 |
| T0 | 48.83（5.20） | 50.00（5.93） |  |  |  |  |  |  |
| T1 | 48.04（5.05） | 51.17（6.24） |  |  |  |  |  |  |
| T2 | 49.31（5.57） | 50.80（6.27） |  |  |  |  |  |  |
| T3 | 49.13（4.59） | 50.25（6.07） |  |  |  |  |  |  |
| CGI-S | | | 5.830 | 0.120 | 1.586 | 0.663 | 0.209 | 0.648 |
| T0 | 2.64 (1.00) | 2.24 (0.76) |  |  |  |  |  |  |
| T1 | 2.38 (0.91) | 2.48 (0.90) |  |  |  |  |  |  |
| T2 | 2.44 (0.89) | 2.44 (0.99) |  |  |  |  |  |  |
| T3 | 2.59 (0.85) | 2.28 (0.77) |  |  |  |  |  |  |
| TESS | | | 0.937 | 0.816 | 0.464 | 0.927 | 0.467 | 0.494 |
| T0 | 7.79 (4.27) | 7.20 (3.30) |  |  |  |  |  |  |
| T1 | 7.67 (3.40) | 7.04 (2.89) |  |  |  |  |  |  |
| T2 | 7.73 (3.37) | 7.30 (2.87) |  |  |  |  |  |  |
| T3 | 7.88 (3.13) | 7.02 (2.67) |  |  |  |  |  |  |
| PSP | | | 6.213 | 0.102 | 4.603 | 0.203 | 0.014 | 0.905 |
| T0 | 68.46 (3.82) | 68.88 (4.11) |  |  |  |  |  |  |
| T1 | 68.88 (4.26) | 68.50 (4.65) |  |  |  |  |  |  |
| T2 | 68.94 (3.92) | 68.46 (5.45) |  |  |  |  |  |  |
| T3 | 68.78 (3.24) | 69.78 (4.79) |  |  |  |  |  |  |
| SHAPS | | | 0.174 | 0.676 | 1.135 | 0.287 | 0.027 | 0.869 |
| T0 | 27.96 (4.39) | 27.88 (5.00) |  |  |  |  |  |  |
| T1 | - | - |  |  |  |  |  |  |
| T2 | - | - |  |  |  |  |  |  |
| T3 | 27.75 (3.60) | 27.39 (5.14) |  |  |  |  |  |  |
| DARS | | | 0.234 | 0.629 | 0.860 | 0.354 | 0.162 | 0.687 |
| T0 | 41.83 (10.59) | 40.40 (11.29) |  |  |  |  |  |  |
| T1 | - | - |  |  |  |  |  |  |
| T2 | - | - |  |  |  |  |  |  |
| T3 | 42.03 (8.87) | 41.04 (10.39) |  |  |  |  |  |  |

CGI-S Clinical Global Impression-Severity, DARS Dimensional Anhedonia Rating Scale, PANSS Positive and Negative Syndrome Scale, PSP Personal and Social Performance Scale, SHAPS Snaith-Hamilton Pleasure Scale, TESS Treatment Emergent Symptom Scale.
