# Supplementary material for: A study on Mahjong intervention to improve cognitive impairment in patients with schizophrenia: a pilot, single-blind, randomized, controlled trial
Source: BMC Psychiatry. 2025 Nov 7;25:1070. doi: 10.1186/s12888-025-07321-1 (PMC12595832; doi:10.1186/s12888-025-07321-1)
Supplement: Supplementary file 3 — Supplementary Material 3. [file 12888_2025_7321_MOESM3_ESM.docx]

**Repeated Measures Analysis**

**PAL Total errors (adjusted)**

**Multiply Std Dev**

**Means of Standard Effect**

**By Effects Deviation Size**

**Term Test Power n N K (σm) (σ) (σm/σ) Alpha Beta**

B1(2) GG F 1.0000 24.5 49 1.0 4.82 0.63 7.614 0.05 0.00

W1(4) GG F 1.0000 24.5 49 1.0 1.85 0.45 4.148 0.05 0.00

B1*W1 GG F 1.0000 24.5 49 1.0 0.91 0.45 2.042 0.05 0.00

n's: 24 25

**PAL Total errors (6 shapes, adjusted)**

**Multiply Std Dev**

**Means of Standard Effect**

**By Effects Deviation Size**

**Term Test Power n N K (σm) (σ) (σm/σ) Alpha Beta**

B1(2) GG F 1.0000 24.5 49 1.0 1.87 0.63 2.958 0.05 0.00

W1(4) GG F 1.0000 24.5 49 1.0 0.52 0.45 1.155 0.05 0.00

B1*W1 GG F 0.6788 24.5 49 1.0 0.19 0.45 0.426 0.05 0.32

n's: 24 25

**SWM Between errors**

**Multiply Std Dev**

**Means of Standard Effect**

**By Effects Deviation Size**

**Term Test Power n N K (σm) (σ) (σm/σ) Alpha Beta**

B1(2) GG F 1.0000 24.5 49 1.0 5.65 0.63 8.930 0.05 0.00

W1(4) GG F 1.0000 24.5 49 1.0 0.79 0.45 1.761 0.05 0.00

B1*W1 GG F 1.0000 24.5 49 1.0 0.95 0.45 2.117 0.05 0.00

n's: 24 25

**SWM Strategy**

**Multiply Std Dev**

**Means of Standard Effect**

**By Effects Deviation Size**

**Term Test Power n N K (σm) (σ) (σm/σ) Alpha Beta**

B1(2) GG F 0.4857 24.5 49 1.0 0.18 0.63 0.281 0.05 0.51

W1(4) GG F 0.7855 24.5 49 1.0 0.21 0.45 0.476 0.05 0.21

B1*W1 GG F 1.0000 24.5 49 1.0 0.54 0.45 1.201 0.05 0.00

n's: 24 25

**RTI Simple accuracy score**

**Multiply Std Dev**

**Means of Standard Effect**

**By Effects Deviation Size**

**Term Test Power n N K (σm) (σ) (σm/σ) Alpha Beta**

B1(2) GG F 0.6545 24.5 49 1.0 0.22 0.63 0.344 0.05 0.35

W1(4) GG F 0.4275 24.5 49 1.0 0.15 0.45 0.324 0.05 0.57

B1*W1 GG F 0.5385 24.5 49 1.0 0.16 0.45 0.368 0.05 0.46

n's: 24 25

**RTI simple reaction time**

**Multiply Std Dev**

**Means of Standard Effect**

**By Effects Deviation Size**

**Term Test Power n N K (σm) (σ) (σm/σ) Alpha Beta**

B1(2) GG F 1.0000 24.5 49 1.0 32.00 0.63 50.590 0.05 0.00

W1(4) GG F 1.0000 24.5 49 1.0 12.16 0.45 27.196 0.05 0.00

B1*W1 GG F 1.0000 24.5 49 1.0 19.54 0.45 43.686 0.05 0.00

n's: 24 25

**RTI simple movement time**

**Multiply Std Dev**

**Means of Standard Effect**

**By Effects Deviation Size**

**Term Test Power n N K (σm) (σ) (σm/σ) Alpha Beta**

B1(2) GG F 1.0000 24.5 49 1.0 35.26 0.63 55.743 0.05 0.00

W1(4) GG F 1.0000 24.5 49 1.0 25.67 0.45 57.393 0.05 0.00

B1*W1 GG F 1.0000 24.5 49 1.0 15.93 0.45 35.616 0.05 0.00

n's: 24 25

**RTI Five-choice accuracy score**

**Multiply Std Dev**

**Means of Standard Effect**

**By Effects Deviation Size**

**Term Test Power n N K (σm) (σ) (σm/σ) Alpha Beta**

B1(2) GG F 0.6545 24.5 49 1.0 0.22 0.63 0.344 0.05 0.35

W1(4) GG F 0.4275 24.5 49 1.0 0.15 0.45 0.324 0.05 0.57

B1*W1 GG F 0.5385 24.5 49 1.0 0.16 0.45 0.368 0.05 0.46

n's: 24 25

**RTI Five-choice reaction time**

**Multiply Std Dev**

**Means of Standard Effect**

**By Effects Deviation Size**

**Term Test Power n N K (σm) (σ) (σm/σ) Alpha Beta**

B1(2) GG F 1.0000 24.5 49 1.0 21.39 0.63 33.823 0.05 0.00

W1(4) GG F 1.0000 24.5 49 1.0 15.20 0.45 33.979 0.05 0.00

B1*W1 GG F 1.0000 24.5 49 1.0 23.01 0.45 51.447 0.05 0.00

n's: 24 25

**RTI five-choice movement time**

**Multiply Std Dev**

**Means of Standard Effect**

**By Effects Deviation Size**

**Term Test Power n N K (σm) (σ) (σm/σ) Alpha Beta**

B1(2) GG F 1.0000 24.5 49 1.0 20.95 0.63 33.118 0.05 0.00

W1(4) GG F 1.0000 24.5 49 1.0 27.09 0.45 60.582 0.05 0.00

B1*W1 GG F 1.0000 24.5 49 1.0 20.70 0.45 46.290 0.05 0.00

n's: 24 25
